# Mr. Whitley's Case of a Damson-stone in the Trachea, and on the Operation of Laryngotomy

**Published:** 1818-02

**Authors:** George Whitley

**Affiliations:** Member of the Roy. Coll. of Surg. Lond. Halton, Cheshire


*To the Editors of the London Medical and Physical Journal.*

GENTLEMEN,

HAVING lately been very successful in the operation of Laryngotomy, and feeling it my duty to offer it to the notice of my professional brethren; I shall be obliged, if you think the detailed case worthy of a place in your Journal, by your inserting it.

Though it presents little or no novelty, yet, perhaps, it may tend to embolden the young surgeon, under similar circumstances, and convince him how little attendant danger may follow very considerable incisions of the larynx and trachea. I remain, gentlemen,

Your's, respectfully,

*Halton, Cheshire;* GEORGE WHITLEY,

*Jan.* 3, 1818. *Member of the Roy. Coll. of Surg. Lond.*

August 26, 1817.—A fine healthy boy, seven years of age, son of a labourer in this village, was brought to me, suffering under a most alarming convulsive cough, wheezing and rattling in the throat, and a much impeded utterance. His attendant informed me, he had been playing with a damson-stone made into a whistle, and had got it into his throat. I was convinced, that the foreign body was either in the larynx or trachea. Thinking that an emetic, by producing a sudden action upon the respiratory muscles, might cause the body to be thrown out of its situation, I gave him ten grains of the sulphas zinci. Excessive vomiting and retching followed, but without the wished-for effect. Only one resource was left for the safety of my patient, and, consequently, I proposed to take out the stone by an incision into the larynx. The friends of the boy, from the vulgar outcry against "cutting the throat," would not allow the operation to be performed.

September 1.—I visited my patient daily since the accident, and he has been taking small, but frequent, doses of laudanum in a mucilaginous mixture. By this medicine, he seems lulled and apparently quite easy during the intermission of the paroxysms; but they occur very frequently in the day, each lasting about ten minutes, and threatening immediate suffocation.

September 6.—My patient is now become quite emaciated, and the paroxysms are more violent and frequent. He often expresses a wish to have the stone cut out, but his parents are still unwilling.

September 8.—Owing to the persuasive influence of a kind-hearted lady in this neighbourhood over the friends of the boy, I was this day desired to perform the necessary operation. With the aid of my assistant, and in the presence of two or three respectable gentlemen in this village, I extracted the stone in the following manner. After placing the boy upon a table, with his head a little elevated by a pillow, I directed the knife from the superior part of the thyroid cartilage, or pomum adami, downwards, and in a strait line to the extent of two inches in length. This incision exposed the inner edges of the sterno-hyoidei muscles. Proceeding very carefully to separate these muscles, I laid bare the thyroid and cricoid cartilages, the upper part of the trachea, and the isthmus of the thyroid gland. Supposing, as is generally the case, that the foreign body was impacted in the superior part of the larynx, I plunged a lancet through the notch of the thyroid cartilage, and, with a probe-pointed bistoury, slit the larynx down to the cricoid cartilage. A paroxysm attacking my patient at this moment, forced a considerable quantity of pus through this slit upon the by-standers, but the stone could not be felt. I concluded it had descended into the trachea, and, the bistoury being again introduced, I divided the cricoid cartilage and several of the upper rings of the trachea, taking care not to wound the thyroid gland.—Here I was again disappointed. Having now ample space, I passed my index-finger up the larynx, and could feel the stone in one of the small recesses of the thyroid cartilage. I dislodged it with a probang, and, forcing it up into the mouth, caused it (unluckily for the satisfaction of the operation to the by-standers,) to take the course of the œsophagus. The immediate cessation of the distressing symptoms convinced me of the removal of the stone. The elasticity of the larynx being sufficient to close itself, the external wound was united by straps of adhesive plaster and roller.

September 9.—A brisk dose of castor-oil was given to my patient, which produced two copious evacuations, and, with them, the damson-stone was discovered, to my complete satisfaction.

September 30.—Wound healed, and boy's health quite renovated. His friends think his voice rather weaker than previously to the accident.

---

*** The above paper is truly valuable; but we think it necessary to apprize the ingenious and candid writer, as well as our readers, of a case which has lately occurred to a young nobleman in France, and which is said not to be uncommon in that part of the world. An ear of bearded wheat, having found its way to the trachea, produced a calamitous issue. Such a case is very likely to have happened where this kind of grain is more generally sowed, and where the corn-fields are usually uninclosed from the high road. Whenever it is suspected, the surgeon must take especial care never to attempt any mode of relief after the operation, but by drawing the substance downward. The necessity of such a measure need not be insisted on. We shall be thankful to any of our readers who can inform us of such a case in a British record. —EDIT.

